# Increased heart rate and blood oxygen extraction are key to the ability of schoolmaster snapper (*Lutjanus apodus*) to swim maximally at high temperatures

**DOI:** 10.1242/jeb.252059

**Published:** 2026-05-11

**Authors:** Fiona P. H. Durnford, Emma S. Porter, Anthony K. Gamperl

**Affiliations:** ^1^ Dept. of Ocean Sciences, Memorial University of Newfoundland and Labrador, St. John's, NL, A1C 5S7, Canada; ^2^ Tropical Marine Ecophysiology Laboratory, Cape Eleuthera Institute, Island School, P.O. Box EL-26029, Rock Sound, Eleuthera, The Bahamas

**Keywords:** Temperature, Exercise, Cardiorespiratory physiology, Oxygen consumption, Thermal tolerance, Aerobic capacity

## Abstract

Consistent with data on other tropical fish species: we previously showed that schoolmaster snapper (*Lutjanus apodus*) exhibited a maximum metabolic rate (MMR) during a critical thermal maximum while swimming (CTS_Max_) test that was 60% higher than during a standard critical thermal maximum (CT_Max_) test; and based on this data suggested that this elevated MMR contributed to swimming fish fatiguing at a temperature only 1.4°C lower than their CT_Max_ (37.5 versus 38.9°C, respectively). However, the mechanism(s) underlying the greater MMR and realistic aerobic scope (AS_R_) in fish given a CTS_Max_ test remained unclear. To answer this question, CT_Max_, *U*_crit_ (critical swimming speed) and CTS_Max_ tests were performed on snapper surgically implanted with Transonic^®^ blood flow probes, and we measured heart rate (*f*_H_), stroke volume (*S*_V_), cardiac output (*Q̇*), oxygen consumption (*Ṁ*_O_2__) and blood oxygen extraction (*E*_O_2__; *Ṁ*_O_2__/*Q̇*). The ∼1.75-fold higher MMR observed snapper when given CTS_max_ versus CT_max_ tests was not explained by a greater contribution of *S*_V_ to *Q̇*. Instead, the higher MMR was primarily driven by a ∼40 beat min^−1^ (25%) higher maximum *f*_H_ and a ∼1.55-fold greater increase in *E*_O_2__. This elevated *E*_O_2__ may reflect enhanced oxygen uptake at the gills (i.e. higher arterial oxygen content) due to the fish engaging in ram ventilation while swimming at elevated temperatures and/or improved blood oxygen extraction. These findings highlight the critical role of *E*_O_2__ in setting tolerance limits under combined thermal and activity-related stressors, and caution against relying solely on *f*_H_ variables (i.e. *f*_H,Max_) as an indicator of thermal tolerance.

## Teaser

**Summary:** Increased heart rate and enhanced blood oxygen extraction allow for a much higher maximum metabolic rate in fishes exposed to an acute increase in temperature while near maximally swimming than when at rest.

## INTRODUCTION

Tropical marine fish represent a large portion of global biodiversity and play essential roles in coral reef ecosystems (Bellwood et al., 2019; Roberts et al., 2002; Sobha et al., 2023). Yet, little is known about their thermal tolerance and physiology when compared with temperate species from the global north (Donelson and Munday, 2012; Nati et al., 2024; Rummer and Munday, 2017). This is concerning because tropical marine species, particularly those near the equator, have evolved in thermally stable environments and may be disproportionately affected by global warming (Nati et al., 2021; Rummer et al., 2014; Tewksbury et al., 2008).

Several methodologies have been used to determine the acute thermal tolerance of fishes, including upper and lower incipient lethal temperature tests (LILT and UILT, respectively) (Beitinger et al., 2000; Fry et al., 1942), critical thermal maximum and minimum tests (CT_Max_ and CT_Min_, respectively) (Becker and Genoway, 1979; Cowles and Bogert, 1944; Lutterschmidt and Hutchison, 1997) and the ‘rapid screening protocol’/‘F_H,Max_ test’ (Casselman et al., 2012; Gilbert et al., 2024). Among these, the CT_Max_ test is the most commonly used, and involves increasing water temperature at a constant rate until loss of equilibrium (LOE). However, its ecological relevance is debated as many studies have used heating rates that greatly exceed those measured in nature (1–4°C h^−1^; Desforges et al., 2023; Gamperl and Stevens, 2026) and this test is performed on resting animals, despite the fact that many fish species actively swim for prolonged periods when engaging in ecologically important behaviours such as schooling, feeding and long-distance migration.

As organ systems that determine performance and are essential to exercise may fail at temperatures much lower than those measured in CT_Max_ tests (Ern et al., 2023; Mayer, 2022; Ørsted et al., 2022), the CTS_Max_ test (previously named the CT_Swim_ test by Blasco et al., 2020) was developed, in which fish constantly swim near their maximal aerobic capacity (i.e. at ∼80–85% of their critical swimming speed, *U*_crit_) while temperature is increased until they fatigue (Blasco et al., 2020). Previous studies have reported that CTS_Max_ is ∼1.5°C lower than the CT_Max_ (Blasco et al., 2020, 2022) for the South American Pacu (*Piaractus mesopotamicus*) and the Nile tilapia (*Oreochromis niloticus*) and 4°C higher (Nati et al., 2023) for the sea bass (*Dicentrarchus labrax*). This was despite values for MMR and AS being 30% higher when measured using the CTS_Max_ method versus during *U*_crit_ or CT_Max_ tests (Blasco et al., 2020; Nati et al., 2023). Furthermore, these authors found that the temperature at which fish started to ‘burst-and-coast’ swim (i.e. where they began to use anaerobically powered muscle contractions) was close to the temperature at which they fatigued (i.e. their upper thermal tolerance), and used this result to infer that a fish's CTS_Max_ is determined by the inability to meet the combined oxygen demands of swimming and warming (Blasco et al., 2020; Nati et al., 2023). These results are in general agreement with the controversial oxygen- and capacity-limited thermal tolerance (OCLTT) hypothesis (Ern et al., 2023; Jutfelt et al., 2018; Pörtner, 2010, Pörtner et al., 2017), which proposes that thermal limits in ectotherms (in this case fatigue) are governed by the inability of the cardiovascular system to deliver sufficient oxygen to the tissues as temperatures rise.

Given the limited data on the swimming and metabolic performance of tropical marine fish species, Nati et al. (2024) performed CT_Max_, *U*_crit_ and CTS_Max_ protocols on ∼26°C-acclimated juvenile (30–40 g) schoolmaster snapper (*Lutjanus apodus*) to assess the relationship between different measures of thermal tolerance and performance. They found that CTS_Max_ (37.5±0.1°C) was only modestly lower than CT_Max_ (38.9±0.1°C), and that maximum metabolic rate (MMR) and realistic aerobic scope (AS_R_; measured as MMR – routine metabolic rate) during the CTS_Max_ test were ∼42% and 65% higher, respectively, compared with values measured in the CT_Max_ and *U*_crit_ tests. These results were very consistent with the findings of Blasco et al. (2020, 2022) and Nati et al. (2023). Interestingly, however, no transition to burst-and-coast swimming occurred during the CTS_Max_ test on schoolmaster snapper, unlike during the *U*_crit_ test, and this finding suggested that insufficient oxygen transport to the tissues was not the primary limiting factor determining thermal tolerance in this test.

While the study by Nati et al. (2024) provided valuable insights into the thermal tolerance of tropical marine fishes, the physiological mechanism(s) that determine temperature-dependent fatigue in the CTS_Max_ test, particularly those related to the increased MMR and AS_R_, remain unresolved. Although heart rate (*f*_H_) is widely recognised as the primary driver of increased cardiac output (*Q̇*) during acute warming, other factors such as stroke volume (*S*_V_), blood oxygenation and oxygen extraction (*E*_O_2__) also play important roles. For instance, in resting fish (as in CT_Max_ tests), *S*_V_ (and thus *Q̇*) may be constrained as temperature increases owing to reduced diastolic filling time and stable (unchanged) central venous pressure (CVP) (Farrell et al., 2009; Sandblom and Axelsson, 2007). In contrast, swimming fish have enhanced venous return, and this can elevate CVP and enable higher *S*_V_ even at elevated heart rates (Sandblom et al., 2005; Sandblom, 2007). Furthermore, recent data suggest that blood oxygen extraction (i.e. the arterio–venous oxygen content difference) is higher in fishes exposed to high temperatures, hypoxia and exercise, and that this supports aerobic performance when *Q̇* is constrained or insufficient to meet metabolic demands (Claësson et al., 2016; Gamperl et al., 2025a; Harter et al., 2019; Keen and Gamperl, 2012; Leeuwis et al., 2021; Motyka et al., 2017).

To explore how the above cardiorespiratory variables are modulated when fish are given *U*_crit_ versus CT_Max_ versus CTS_Max_ tests, we tested three hypotheses in schoolmaster snapper fitted with Transonic^®^ flow probes for the measurement of cardiac function: (1) an enhanced *S*_V_ in swimming fish (i.e. during a CTS_Max_ test) is responsible for, or contributes to, the higher MMR and AS_R_ in these fish in comparison to those given a CT_Max_ test (i.e. where the fish are resting); (2) a temperature-dependent increase in maximum heart rate (i.e. *f*_H,max_), and thus scope for *f*_H_, is responsible for the higher MMR and AS_R_ observed in fish given a CT_Max_ versus *U*_crit_ test; (3) enhanced blood oxygen extraction (*E*_O_2__; *Ṁ*_O_2__/*Q̇*) during a CTS_Max_ test contributes to the increased MMR and AS_R_ in snapper as compared with fish given a CT_Max_ test.

The schoolmaster snapper is a widely distributed tropical/subtropical marine species that in the Bahamas experiences relatively small diel and seasonal changes in temperature (∼4–6°C), and engages in sustained aerobic activities such as shoaling, foraging and predator avoidance (The Online Guide to the Animals of Trinidad and Tobago; Macdonald et al., 2009; Szekielda, 2024). As such, it is a relevant model for examining how integrated cardiovascular and respiratory responses support fish aerobic capacity during acute warming, sustained exercise, and when these two metabolically demanding challenges are combined.

**Table:** 

**List of symbols and abbreviations**	
ANOVA	analysis of variance
AS	aerobic scope
AS_R_	realistic aerobic scope
BL	body length
CEI	Cape Eleuthera Institute
CT_Max_	critical thermal maximum
CT_Min_	critical thermal minimum
CTS_Max_	critical thermal maximum while swimming
CT_Swim_	temperature at which a fish fatigues when swimming
CVP	central venous pressure
*E*_O_2__	blood oxygen extraction
*f*_H_	heart rate
*f*_H,Max_	maximum heart rate
h	hour
LILT	lower incipient lethal temperature
LOE	loss of equilibrium
MMR	maximum metabolic rate
*Ṁ*_O_2__	oxygen consumption
OCLTT	oxygen and capacity limited thermal tolerance
O_2_	oxygen
*Q̇*	cardiac output
RMR	resting metabolic rate
ROUT	robust regression and outlier removal
RVM	relative ventricular mass
SMR	standard metabolic rate
*S*_V_	stroke volume
TMS	tricaine methanesulphonate
*U*_crit_	critical swimming speed
UILT	upper incipient lethal temperature

## MATERIALS AND METHODS

### Ethical approval

This study received ethical approval from the Animal Care Committee at Memorial University of Newfoundland and Labrador (Protocol No. 24-01-KG) and was approved by the Departments of Marine Resources and Environmental Protection and Planning of The Bahamas (DEPP Research Permit No. BS-2023-522210). All procedures involving fish were conducted in full compliance with the Canadian Council on Animal Care's guidelines for the care and use of fish in research, teaching and testing (CCAC, 2005). It should be noted that all fish were only tested once (i.e. they were not utilised in multiple protocols).

### Fish husbandry

Subadult schoolmaster snapper [*Lutjanus apodus* (Walbaum 1792); ∼200–300 g; 17–20 cm] were caught in the spring and early summer of 2024 (1 May to 5 July) using baited wire traps in the vicinity of the Cape Eleuthera Institute (CEI; Eleuthera, The Bahamas). Once captured, the fish were transported in aerated seawater to CEI where they were held outdoors in 1.3 m^3^ round tanks for no longer than 2 weeks prior to experimentation. Tanks were supplied with well-aerated seawater (∼34 ppt and >95% air saturation) at ambient temperatures of 26–28°C. The fish were fed *ad libitum* daily with frozen sardines (*Sardinella aurita*) and conch (*Aliger gigas*) to which a vitamin mix (VitaChem Marine Formula; Boyd Enterprises, Miami, Florida, USA) and vitamin E drops (Boyd Enterprises) had been added. Fish were fasted for 24 h prior to surgery.

### Surgical procedures

Fish were netted from their holding tank and anaesthetised in aerated seawater containing tricaine methanesulfonate (TMS, 0.2 g l^−1^; Syndel Laboratories Ltd, Qualicum Beach, BC, Canada) buffered with an equal amount of sodium bicarbonate until ventilatory movements ceased. Once the fish's mass (g), fork length (cm) and girth (cm) were measured, they were placed on their right side on a wetted foam pad upon a surgical table where their gills were continuously irrigated with buffered and oxygenated seawater containing a maintenance dose of TMS (0.1 g l^−1^) at tank temperature. To allow access to the opercular cavity, umbilical tape was passed beneath the gill arches and tied to the operating table. A small puncture in the skin of the opercular cavity (just below the junction of the second and third gill arches) was then made using a pair of Dumont forceps. The ventral aorta was located and freed of connective tissue using blunt dissection, ensuring that the pericardium remained intact/was not damaged. A custom temperature-calibrated Transonic^®^ flow probe (Model PS Transducer 1.5–2.0 mm; Transonic^®^ Systems, Ithaca, NY, USA) was fitted around the ventral aorta (Gollock et al., 2006; Leeuwis et al., 2021), and the probe leads were connected to a Transonic^®^ flow meter (model T402; Transonic Systems) to ensure a good signal and flow profile. Finally, the probe leads were secured to the fish at three locations using 1–0 silk suture: (1) to the ceratobranchial element lining the posterior margin of the fourth buccal-opercular opening which is absent of gill filaments; (2) just below the lateral line; and (3) just anterior to the dorsal fin (Leeuwis et al., 2021; Porter and Gamperl, 2023; Sandrelli and Gamperl, 2023).

### Recovery and equipment

Following the completion of surgery (which took approximately 15–25 min), fish selected for CT_Max_ testing were placed into one of two cylindrical respirometers (*N*=2 per trial) (∼7.0 l each; 14 cm in diameter and 45 cm in length) submerged in a raceway with 28°C water; each respirometer equipped with flush and recirculating pumps (both 5 l min^−1^ Eheim, Model 1046; Eheim Gmbh and Co., Deizisau, Germany). For approximately 20 h (until the following morning), water was circulated through the chambers using the flush pump, and oxygen levels in the water were maintained at >95% air saturation by bubbling air directly into the water in the raceway. The respirometers were equipped with calibrated (using air saturated water and water with 50 mmol l^−1^ sodium sulphite) dipping probes that were connected to a FireSting fibre-optic oxygen meter (Pyroscience^®^, Archen, Germany), and the meter's output was recorded on a computer running Pyro Oxygen Logger software (Pyroscience^®^). The flush pump and recirculation pump were controlled by Smart Shifter software (Norin and Gamperl, 2018), and were intermittently turned on or off to either flush the respirometer with fresh seawater or to create a sealed respirometry chamber when off. Finally, in this setup, temperature was controlled using an 1800 W programmable immersion heater (Intelligent Heater, Clepco, GA, USA).

Fish selected for *U*_crit_ and CTS_Max_ protocols were put in a 108.7 l Bläzka-type swim-tunnel respirometer (University of Waterloo, Biotelemetry Institute, Waterloo, ON, USA), with an internal diameter of 24 cm and a 100 cm long working section, filled with ambient seawater at 28°C (i.e. at the same temperature as the holding tank). The front of the swim tunnel was fitted with a plastic grid that allowed for uniform water flow in the swimming section of the tunnel (Taylor and McPhail, 1985). The front of the swim tunnel was also covered with black plastic to provide the fish with a dark refuge and to minimise stress from external stimuli. After ∼6 h of recovery from surgery, the water velocity was set to 0.25 body lengths (BL) s^−1^ and the fish left overnight to recover and acclimatise to the swim tunnel. In this setup, water was delivered to the swim tunnel from a 200 l reservoir using a 10 l min^−1^ (Little Giant model NK-1; Franklin Electric, Fort Wayne, IN, USA) submersible pump, and temperature and oxygen levels in the reservoir were controlled by programmable immersion heaters (Intelligent Heater-Clepco) and air stones supplied with air or pure oxygen, respectively. The latter was required to maintain the water O_2_ level in the swim tunnel at >95% when the fish were swimming at high temperatures. Oxygen consumption measurements were initiated by manually stopping the flow of water into and out of the swim tunnel, and measuring the drop in water O_2_ levels using a pre-calibrated fibre-optic dipping probe connected to a PreSens O_2_ meter (Fibox3 LCDV3 version 2.0.1.0; PreSens Precision Sensing GmBH; Regensburg, Germany) interfaced with a laptop computer running PreSens PST3 version 520 software.

### Experiments

The lights in the lab were turned on at 07:00 h, and the flow probe leads were connected to the Transonic^®^ flow meter. Approximately 1 h after entering the lab, basal (resting) cardiorespiratory variables (*Q̇*, *f*_H_, *S*_V_ and *Ṁ*_O_2__) were measured, and the fish were subjected to a CT_Max_, *U*_crit_ or CTS_Max_ test (*N*=8–9 per group). Upon completion of all experiments, background *Ṁ*_O_2__ was measured. However, this variable was found to be negligible (<1%), confirming that microbial respiration did not contribute significantly to the measured values of oxygen consumption (Rodgers et al., 2016; Svendsen et al., 2016a).

### Critical thermal maximum (CT_Max_) test

To determine the snapper's acute upper thermal tolerance, we used a CT_Max_ test (Desforges et al., 2023), which involved gradually increasing water temperature in the raceway/respirometer by 2°C h^−1^ until the fish lost equilibrium. Cardiorespiratory variables (*Q̇*, *f*_H_, *S*_v_ and *Ṁ*_O_2__) were recorded at every 1°C increment (detailed below). These measurements were taken during the closed portion of the respirometry cycle, which was 10–15 min depending on temperature.

### Critical swimming speed (*U*_crit_) test

Cardiorespiratory and metabolic variables were recorded in all fish during a *U*_crit_ test (Brett, 1964). Resting (routine) cardiac function and *Ṁ*_O_2__ were measured at a baseline speed of 0.25 BL s^−1^, where the fish could maintain position without actively swimming. The speed was then increased to 0.5 BL s^−1^ to initiate continuous swimming, followed by increments of 0.25 BL s^−1^ every 20 min until they became exhausted (i.e. they were unable to move off the tunnel's rear grid in 10 s). Following fatigue, the water velocity was reduced to baseline (0.25 BL s^−1^) for 1 h prior to euthanasia. Note that all swimming velocities and calculations of *U*_crit_ were corrected for solid blocking effects (Bell and Terhune, 1970; Kline et al., 2015) using the following equations:
(1)

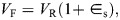
where *V*_F_ is the water velocity at the position of the fish's maximum girth; *V*_R_ is the water velocity at the rear of the flume and ∈_S_ is the error due to solid blocking, which was calculated as:
(2)

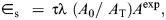
where τ is a dimensionless factor for tunnel cross-sectional shape (0.8); λ is a factor (coefficient) for the shape of the fish, set at 0.5 (i.e. this value for a fish with streamlined shape); *Α*_0_ is the cross-sectional area of the fish, calculated as 0.25 *G*^2^ π^−1^, where *G* is the maximum girth to the closest mm; *A*_T_ is the cross-sectional area of the swimming chamber, calculated as π*r*^2^, where the radius (*r*) was 125 mm; and *A*^exp^ is the fractional area exponent (1.5) (Kline et al., 2015). The fish's critical swimming speed was calculated as:
(3)

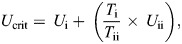
where *U*_i_ is the velocity at which the fish swam for the entire time increment; *U*_ii_ is the velocity increment (0.25 BL s^−1^); *T*_i_ is the time elapsed from the last change in current velocity to fatigue; and *T*_ii_ is the time increment (i.e. the time between increases in velocity, 20 min).

### Critical thermal maximum while swimming (CTS_Max_) test

Before conducting CTS_Max_ trials, preliminary experiments confirmed that fish could sustain swimming for at least 5 h at 75% of their *U*_crit_, as determined in experiment 2. This threshold was selected instead of the 80–85% *U*_crit_ used by Nati et al. (2023, 2024) and Blasco et al. (2022), as fish in the current study were unable to maintain swimming at the higher speed. This was likely due to the effects of the surgical procedures and/or the drag of the flow probe leads on fish swimming performance (see discussion). In the CTS_Max_ trials, water velocity was initially increased to 75% of the schoolmaster snapper's *U*_crit_ over 1 h; this speed was maintained for 20 min at 28°C so that cardiorespiratory variables could be measured, and then water temperature was increased at 2°C h^−1^ until the fish fatigued. Cardiorespiratory variables were recorded at every 1°C increment and the temperature at fatigue was recorded as the fish's CTS_Max_. Upon fatigue, water velocity and temperature were immediately reduced to 0.25 BL s^−1^ and 28°C, respectively, and the fish was allowed to recover for 1 h prior to being euthanized.

### Data and statistical analyses

After the experiments were completed, all fish were euthanised using 0.4 g l^−1^ TMS. The ventricle was removed and weighed, and relative ventricular mass (RVM) was calculated as: (ventricular mass/fish mass)×100. The signals from the Transonic^®^ flow meter were filtered and amplified by a data acquisition system (MP160; BIOPAC Systems, Santa Barbara, CA, USA) with a universal interface module (UIM100A, BIOPAC Systems, Goleta, CA, USA) and recorded on a computer running AcqKnowledge^®^ software (version 5.0; BIOPAC Systems).

Heart rate (*f*_H_) was calculated by averaging the time required for 20 peaks to appear in two separate blood flow traces and stroke volume (*S*_V_) was calculated as *Q̇*/*f*_H_. Oxygen consumption (*Ṁ*_O_2__) was calculated by measuring the slope of the relationship between the water's oxygen level (in mg l^−1^) and time, after allowing for a 2 min wait period. MMR was recorded as the maximum *Ṁ*_O_2__ value recorded for each of the three protocols (although they differed significantly), and realistic aerobic scope (AS_R_) and factorial aerobic scope (FAS) were calculated as MMR–RMR and MMR/RMR, respectively. Finally, blood oxygen extraction (*E*_O_2__; the amount of oxygen consumed per mL of blood pumped) was calculated as:
(4)

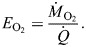


All statistical analyses, including that of the morphometric variables [mean weight (g), length (cm), condition factor (K) and RVM], were conducted using GraphPad Prism (version 10.2.0, GraphPad Software, San Diego, CA, USA). Outliers were identified using the ROUT method (robust regression and outlier removal) with a Q value of 1%, which controls the maximum desired false discovery rate. Identified outliers were excluded from subsequent analysis. The data were then tested for assumptions of normality and homogeneity of variance using the Shapiro–Wilks and Levene's tests, respectively. One-way ANOVAs, followed by Tukey's *post hoc* tests, were used for all statistical comparisons, with the level of significance set at *P*<0.05, and in cases where assumptions were not met (i.e. resting and maximum *S*_V_), non-parametric Kruskal–Wallis tests, followed by Dunn's *post hoc* tests, were used.

## RESULTS

Resting *Ṁ*_O_2__ was significantly lower (by ∼25%; ∼175 versus 220 mg O_2_ kg^−1^ h^−1^) in the fish that were placed in the static respirometers (i.e. given the CT_Max_ test) compared with those that were recovered in the Blazkä swim tunnel [i.e. those given *U*_crit_ and CTS_Max_ tests (Table 1)]. Furthermore, although there were no differences in resting *f*_H_ or *Q̇* between the groups, *S*_V_ and *E*_O_2__ were ∼20% lower and 43% higher, respectively, in snapper subjected to CTS_Max_ and *U*_crit_ tests compared with those given a CT_Max_ test (Table 1).

**Table 1.** Cardiorespiratory variables for schoolmaster snapper when subjected to a critical swimming speed (*U*_crit_) test, a critical thermal maximum (CT_Max_) test, and a critical thermal tolerance while swimming (CTS_Max_) test

	Test	Resting value	Maximum value	Absolute scope	Factorial scope
*Ṁ*_O_2__ (mg O_2_ kg^−1^ h^−1^)	*U*_crit_	224.92±10.41^a^	684.57±20.26^a^	459.66±19.06^a^	3.08±0.14^a,b^
	CT_Max_	176.42±11.96^b^	448.96±22.63^b^	272.53±23.59^b^	2.62±0.28^b^
	CTS_Max_	214.68±12.70^a^	781.31±16.50^c^	566.64±19.47^c^	3.73±0.23^a^
*Q̇* (ml min^−1^ kg^−1^)	*U*_crit_	28.54±2.02^a^	58.48±3.44^a^	29.94±2.47^a,b^	2.07±0.11^a,b^
	CT_Max_	32.56±2.02^a^	61.00±3.56^a^	28.44±3.92^a^	1.91±0.15^a^
	CTS_Max_	28.26±1.01^a^	69.12±2.39^a^	40.87±2.94^b^	2.48±0.15^b^
*f*_H_ (beats min^−1^)	*U*_crit_	96.41±7.05^a^	153.01±3.74^a^	56.60±4.21^a^	1.63±0.17^a^
	CT_Max_	86.13±5.78^a^	145.14±8.48^a^	59.01±7.76^a^	1.72±0.12^a,b^
	CTS_Max_	90.30±3.58^a^	183.09±3.72^b^	92.79±5.48^b^	2.05±0.10^b^
*S*_V_ (ml kg^−1^)	*U*_crit_	0.306±0.030^a^	0.429±0.030^a^	0.123±0.010^a^	1.43±0.06^a^
	CT_Max_	0.383±0.018^b^	0.472±0.028^a^	0.089±0.023^a^	1.24±0.07^a^
	CTS_Max_	0.316±0.015^a^	0.425±0.016^a^	0.109±0.019^a^	1.36±0.07^a^
*E*_O_2__ (mg O_2_ ml^−1^ blood)	*U*_crit_	0.134±0.01^a^	0.205±0.013^a^	0.071±0.011^a,b^	1.54±0.08^a^
	CT_Max_	0.091±0.01^b^	0.134±0.004^b^	0.043±0.015^a^	1.52±0.12^a^
	CTS_Max_	0.126±0.01^a^	0.207±0.015^a^	0.081±0.014^b^	1.65±0.10^a^

Resting values, maximum values and the absolute and factorial scope for metabolic rate (*Ṁ*_O_2__)_,_ heart rate (*f*_H_), cardiac output (*Q̇*), stroke volume (*S*_v_) and blood oxygen extraction efficiency (*E*_O_2__) are shown. All values are means±s.e.m, *N*=8 per group. Superscript letters (a,b,c) indicate groups that differ significantly from one another (*P*<0.05).

**Fig. 1.**
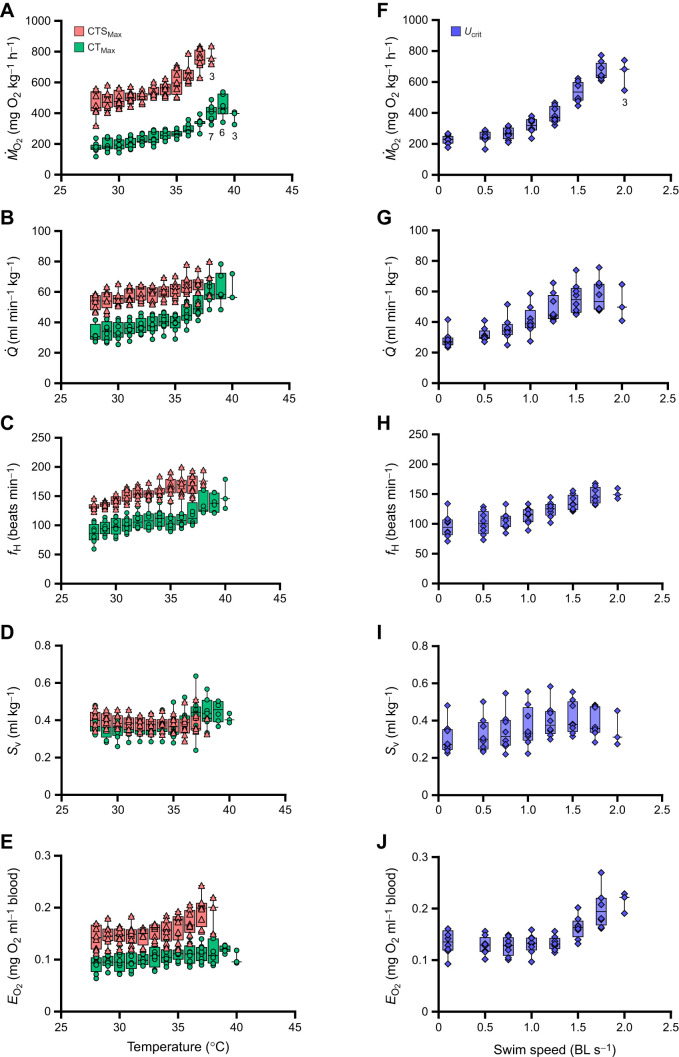
**Cardiorespiratory variables measured in schoolmaster snapper during the three experimental protocols.** Critical thermal maximum (CT_Max_), critical thermal maximum while swimming (CTS_Max_; A–E) and critical swimming speed (*U*_crit_; F–J) tests were performed. (A,F) Changes in oxygen consumption (*Ṁ*_O_2__). (B,G) Changes in cardiac output (*Q̇*). (C,H) Changes in heart rate (*f*_H_). (D,I) Changes in stroke volume (*S*_v_). (E,J) Changes in blood oxygen extraction (*E*_O_2__). Lower and upper box boundaries indicate the 25th and 75th quartiles, respectively; the line inside the box is the median value, and the upper and lower ‘whiskers’ indicate the highest and lowest values. *N*=8–9 per group.

*Ṁ*_O_2__ increased in a curvilinear fashion irrespective of whether the fish were exposed to: (1) an increase in temperature (CT_Max_ test); (2) an increase in swimming speed to exhaustion (*U*_crit_ test); or (3) an increase in temperature while swimming at 75% of their *U*_crit_ (CTS_Max_ test) (Fig. 1). However, there were large differences in MMR, AS_R_ and FAS between the groups, with values for fish given the CTS_Max_ test>*U*_crit_ test>>CT_Max_ test (*P*=0.001; Table 1). For example: (1) fish given the CTS_Max_ test had a MMR of ∼780 mg O_2_ kg^−1^ h^−1^ compared with 685 mg O_2_ kg^−1^ h^−1^ and 450 mg O_2_ kg^−1^ h^−1^ in fish given the *U*_crit_ and CT_Max_ tests, respectively; and (2) AS_R_ and FAS for the three groups were ∼566, 460 and 272 mg O_2_ kg^−1^ h^−1^ and 3.73, 3.08 and 2.62, respectively (Table 1).

Maximum *Q̇* was numerically (i.e. ∼70 versus 58–61 ml kg^−1^ min^−1^), but not statistically (*P*>0.05), higher in fish given the CTS_Max_ test compared with both of the other groups. In addition, the absolute and factorial scopes for *Q̇* were both greater in the CTS_Max_ group compared with the CT_Max_ group (by ∼45 and 30%, respectively) (Table 1). This was entirely due to significantly higher values for maximum *f*_H_ (183 versus 153 and 145 beats min^−1^), and absolute (∼93 versus 58 beats min^−1^) and factorial scope (∼2.1 versus 1.7) for *f*_H_ in fish given the CTS_Max_ versus the CT_Max_ and *U*_crit_ tests, respectively. Stroke volume increased in all groups by a similar amount (∼25–40%). Although *E*_O_2__ values were not different between fish given the CTS_Max_ versus *U*_crit_ tests (and thus the higher *f*_H_ and *Q̇* values likely explain the 14% higher MMR in the CTS_Max_ group), *E*_O_2__ was clearly constrained in snapper given the CT_Max_ protocol compared with the two other tests. Values for maximum *E*_O_2__, and the absolute scope for *E*_O_2__, were ∼40 and 70% greater, respectively, in fish given the CTS_Max_ and *U*_crit_ protocols compared with the CT_Max_ test (Table 1).

As part of these experiments, we were able to determine the schoolmaster snapper's swimming performance and upper thermal tolerance. The *U*_crit_ of the schoolmaster snapper was 2.04±0.07 BL s^−1^ (body lengths per second) and was reached after the fish showed burst-and-coast swimming behavior. The CT_Max_ value for these fish was 39.7±0.3°C, and this value was only ∼1.7°C higher than measured in the CTS_Max_ protocol (38.0±0.10°C). In contrast to the *U*_crit_ test, the schoolmaster snapper did not engage in burst-and-coast swimming prior to reaching their CTS_Max_.

## DISCUSSION

Building on the findings of Nati et al. (2024), this study examined the physiological mechanisms responsible for the elevated MMR and AS_R_ previously observed in juvenile schoolmaster snapper (*Lutjanus apodus*) during a CTS_Max_ challenge as compared with CT_Max_ and *U*_crit_ tests. We found that the much greater MMR during the CTS_Max_ test was primarily due to higher values for maximum *f*_H_ and *E*_O_2_ _and not differences in *S*_V_. This study represents, to the best of our knowledge, the first direct assessment of how heart function and oxygen extraction of a tropical fish species respond to the combined stressors of high temperature and near-maximal aerobic exercise. These findings contribute to a better understanding of how physiological plasticity might shape the resilience of fishes in the face of climate change, as performance-temperature interactions are central to defining these limits (Johansen et al., 2021; Munday et al., 2008; Rummer et al., 2014). Specifically, these data add to recent evidence supporting the importance of *E*_O_2__ in determining fish temperature-dependent metabolic capacity (see below) and raise a critical new question. What mechanism(s) allow snapper during a CTS_max_ test to achieve values of *f*_Hmax_ that are well above those in fish exposed to temperature or maximal swimming challenges alone?

### Resting cardiorespiratory function

The resting *Ṁ*_O_2__ of schoolmaster snapper during the CT_Max_ test (∼175 mg O_2_ kg^−1^ h^−1^) was similar to values reported in previous CT_Max_ experiments conducted on the same species in respirometers at comparable acclimation temperatures (e.g. Sandrelli et al., 2024). All fish in this study were instrumented with ventral aortic flow probes; however, those that recovered in the Bläzka-type swim tunnel, and were subsequently used for *U*_crit_ and CTS_Max_ tests, had resting *Ṁ*_O_2__ values approximately 25% higher (∼220 mg O_2_ kg^−1^ h^−1^). This discrepancy in RMR may have arisen from subtle experimental differences. For instance, low but non-zero flow in a swim tunnel could require fish to maintain position or ventilate differently, which has been noted as a potential source of variation in respirometry measurements (Steffensen, 1989). In addition, the presence of external instrumentation (e.g. a flow probe lead) may have introduced slight drag or mechanical tension, which, although not widely quantified, represents a potential source of additional metabolic cost under some respirometry conditions (Chabot et al., 2016; Svendsen et al., 2016b). These findings indicate that even under nearly identical experimental conditions, RMR can be sensitive to small differences in the circumstances under which measurements are performed.

With respect to the mechanisms that mediated these differences in RMR, it was apparent that the fish in the respirometers had a significantly greater *S*_V_ (and quantitatively higher *Q̇*, by 15%) at rest, whereas the snapper in the swim tunnel had higher values for *E*_O_2_ _(Table 1). The reasons for this are not obvious. However, the slight current in the swim tunnel may have improved water flow over the gills and gas-exchange efficiency, resulting in the higher *E*_O_2__ at rest. Such context-dependent shifts between perfusion- and diffusion-driven oxygen delivery have been observed in fishes under varying hydrodynamic and environmental conditions (Gamperl et al., 2025a; Korsmeyer, 1996; McArley et al., 2022). Collectively, these findings emphasise that the physiological baseline preceding a thermal challenge is shaped not only by temperature and acclimation conditions, but also by subtle differences between experimental setups and protocols. This is important as it has been suggested that resting (basal) physiological functions, but not maximum performance capacities, can adjust to warming conditions (i.e. the ‘plastic floors, concrete ceilings’ hypothesis; Sandblom et al., 2016), and thus it is critical that estimates of standard metabolic rate (SMR) and RMR are accurate.

### Comparison of *U*_crit_, CT_max_ and CTS_max_ values with the literature

The critical swimming speed (*U*_crit_) of *L. apodus* (2.04±0.07 BL s^−1^) in this study is comparable to values reported for similarly sized schoolmaster snapper and other moderately active reef fishes (Fulton, 2007; Malorey et al., 2024; Nati et al., 2024; Plaut, 2001; Schneider et al., 2023). This positions the schoolmaster snapper as a competent demersal reef predator, capable of preying on both benthic invertebrates when small and fishes when larger, consistent with its benthopelagic lifestyle (Rooker, 1995; Potts et al., 2016). Morphology and activity level are key determinants of swimming endurance and aerobic capacity in reef taxa (George and Westneat, 2019; Killen et al., 2016; Metcalfe et al., 2016; Rubio-Gracia et al., 2020), and the streamlined body and moderate activity level of *L. apodus* probably support an intermediate performance between sedentary coral dwellers (e.g. the Nassau grouper; Porter and Gamperl, 2023) and highly active pelagic species (e.g. see Table 1 in Gamperl et al., 2025b for species' comparisons).

The CTS_Max_ protocol combines near-maximal swimming and acute warming to assess the upper thermal tolerance of fishes based on the end point of fatigue. In *L. apodus*, CTS_Max_ was approximately 1.7°C lower than CT_Max_ (38.0 versus 39.7°C, respectively) and this value is very similar to the ∼1.5°C difference reported for other tropical fish species (Blasco et al., 2020, 2022; Nati et al., 2023, 2024). Importantly, these data suggest that while CT_Max_ tests are frequently used to estimate the upper thermal limits of fishes, and remain valuable for standardising comparisons of acute thermal tolerance between species and life stages (e.g. see Desforges et al., 2023; Gamperl and Stevens, 2026 results), they may not accurately reflect the thermal tolerance or aerobic capacity of fishes if they encounter high temperatures while actively engaged in swimming (i.e. CT_Max_ for many species may overestimate thermal tolerance under ecologically relevant activities such as migration, and feeding and predator avoidance). Interestingly, Nati et al. (2024) raise the possibility that the benefits of ram ventilation at intermediate swimming speeds may result in an upper thermal tolerance that is even higher than CT_Max_, and we question the use of this metric of thermal tolerance for many fishes (Gamperl and Stevens, 2026 results). For most fish, swimming at maximum aerobic capacity for prolonged periods represents a small proportion of their life history, and assessment of their thermal tolerance at ecologically-relevant swimming speeds might be more beneficial for conservation and management purposes.

### Mechanistic basis of the observed differences in metabolic capacity

Fish given the CTS_Max_ test had much higher MMR and AS_R_ values compared with those given the CT_Max_ and *U*_crit_ tests, and the increase in these variables was associated with higher values for *f*_H_ and *E*_O_2__, but not *S*_V_ (Fig. 1 and Table 1). This lack of an increase in *S*_V_ is in contrast to our original hypothesis, and surprising, given that Steinhausen et al. (2008) showed that adult sockeye salmon swum at 75% of their *U*_crit_ at temperatures up to 23°C were able to maintain *S*_V_ at twice the level at rest and Sandblom et al. (2005) showed that central filling pressure (CVP; i.e. the heart's filling pressure) increases in swimming sea bass (*Dicentrarchus labrax*) at 22°C. However, in the schoolmaster snapper, only *f*_H_ was higher in swimming compared with resting fish at their acclimation temperature, and *S*_V_ increased by a similar degree (24%) in fish given the CT_Max_ test compared with the other two tests. This latter response is atypical (see Eliason and Anttila, 2017), and questions whether tropical fishes respond differently to acute increases in temperature compared with temperate and polar species. Furthermore, increased cardiac preload does not necessarily lead to an increased *S*_V_ in fishes, and might have only allowed the schoolmaster snapper to maintain, and not increase, *S*_V_ at the very high heart rates observed in snapper during the *U*_crit_ and CTS_Max_ tests (see below). These *f*_H_ values are much higher than those observed in Steinhausen et al. (2008) for their swimming salmon (∼105 beats min^−1^).

Maximum *f*_H_ was 183 beats min^−1^ during the CTS_Max_ test, and this value was significantly higher than the 153 beats min^−1^ observed for fish given a CT_Max_ test and the 145 beats min^−1^ recorded for fish given the *U*_crit_ test. Based on these differences, we would have expected that fish given the CTS_Max_ test would also have a greater maximum or scope for *Q̇*, and indeed scope for *Q̇* was significantly higher in CTS_Max_ versus CT_Max_ fish and higher (but not significantly, *P*>0.05) versus *U*_crit_ fish by ∼20–30%. Thus, these data support our initial hypothesis that a higher *f*_H_ during the CTS_Max_ protocol contributed to the greater values for MMR and AS_R_ in fish given this test. Heart rate in fish is controlled by five main factors: (1) the heart's intrinsic pacemaker rate (rhythm); (2) cholinergic nervous tone which slows *f*_H_ (i.e. has a negative chronotropic effect); (3) humoral and nervous adrenergic tone which increases *f*_H_ (i.e. has a positive chronotropic effect); (4) temperature effects on the pacemaker cell's rate of firing; and (5) cardiac stretch, which increases with *S*_v_ (Anttila et al., 2023; Stoyek et al., 2022; Vornanen, 2017). However, there was no difference in the snapper's *f*_H_ before the tests began (i.e. at acclimation temperature). Furthermore, temperatures during the CT_Max_ and CTS_Max_ tests, and values for *S*_v_ (and thus stretch of the sinoatrial region) were similar. This information points to differences in adrenergic and/or cholinergic tone on the heart being responsible for the higher *f*_H_ values for fish given the CTS_Max_ test. That differences in the amount of cholinergic tone on the heart between fish given the CTS_Max_ test versus the other two tests contributed to the greater *f*_H_ in the former test is quite possible (and very likely). Although data on salmonids (fish with coronary arteries) is contradictory (Ekström et al., 2014, 2021; Gilbert et al., 2019), data on the yellow perch (*Perca fluvacens*) and the roach (*Rutilus rutilus*) (which like *L. apodus* do not have coronary arteries, pers. observation) show that blocking cholinergic tone during a CT_Max_ test results in increases in maximum *f*_H_ of 28 and 47 beats min^−1^, respectively (Ekström et al., 2021). Indeed, these differences in maximum *f*_H_ are in the same range as for the schoolmaster snapper given a CTS_Max_ versus a CT_Max_ test (38 beats min^−1^). However, it does not appear that a greater adrenergic tone on the heart could have resulted in the higher *f*_H_ in snapper given the CTS_Max_ test given that the above studies (which also blocked adrenergic tone on the heart using propranolol and/or sotalol) were unable to demonstrate that this pharmacological intervention affected peak (maximum) *f*_H_.

Enhanced *E*_O_2__ during the CTS_Max_ test also contributed substantially to the higher MMR when compared with fish given the CT_Max_ test, thereby supporting the third hypothesis. However, maximum *E*_O_2__ and the scope for *E*_O_2__ were not different between fish given the CTS_Max_ and *U*_crit_ tests (Table 1). This indicates that it was forced exercise (swimming) alone, and not the combined effects of high temperature and swimming, that resulted in the ∼twofold higher maximum *E*_O_2__ in fish given the CTS_Max_ versus CT_Max_ tests. This enhancement in *E*_O_2__ probably reflects the combined effects of exercise-driven changes in ventilation, circulation and tissue-level gas exchange, which together elevate O_2_ uptake and delivery during periods of high metabolic demand (Claireaux and Lefrançois, 2007; Korsmeyer, 1996; Kiceniuk and Jones, 1977). Exercise-induced acidosis shifts the haemoglobin–oxygen dissociation curve to the right, facilitating oxygen unloading at the tissues (Farrell and Clutterham, 2003; Milligan and Wood, 1986; Nikinmaa and Salama, 1998; Steinhausen et al., 2008), and importantly, ram ventilation during sustained swimming would improve branchial oxygen uptake and help maintain arterial oxygen saturation; at least partially offsetting any effects of these two factors on haemoglobin oxygen loading at the gills (Keen and Gamperl, 2012). Finally, the activity of plasma-accessible carbonic anhydrase (PaCA) at the blood-tissue interface may enhance the conversion of HCO_3_^−^ to CO_2_ and facilitate greater diffusive O_2_ conductance at the tissues, and this mechanism has previously been shown to be important for oxygen uptake in Atlantic salmon (*Salmo salar*) both at rest and during moderate swimming (Harter et al., 2019). However, to what extent these possible mechanisms, and differences in stress-induced catecholamine levels, contributed to the enhanced *E*_O_2__ in fish during the CTS_Max_ test awaits further study.

### Summary and perspectives

This study provides a mechanistic understanding of how combined swimming and thermal stress influence aerobic performance and thermal limits of the schoolmaster snapper. By integrating various cardiorespiratory variables (*Ṁ*_O_2__, *Q̇*, *f*_H_, *S*_V_ and *E*_O_2__), we demonstrate that the substantially higher MMR observed during a CTS_Max_ test (compared with CT_Max_ and *U*_crit_ tests) arises from increases in both maximum *f*_H_ and *E*_O_2__, rather than *S*_V_. These results also show that *E*_O_2__ (a variable rarely reported during acute thermal challenges) can play a central role in determining fish oxygen consumption (in contrast to existing dogma; e.g. see Farrell, 2009) and provides additional evidence that this variable may be particularly important for determining the metabolic capacity of fishes when exposed to multiple stressors, such as exercise and high temperature, or high temperature and hypoxia (Claësson et al., 2016; Motyka et al., 2017; Leeuwis et al., 2021; Gamperl et al., 2025a). Furthermore, our results highlight the physiological flexibility (plasticity) that fishes have to deal with environmental challenges, and that this may be a key feature of their resilience in the face of climate change. For example, one could easily hypothesize that fish swimming at near maximal capacity would have an acute thermal tolerance considerably lower than that in resting fishes; however, this study and those of Blasco et al. (2020, 2022) show that the CTS_Max_ of tropical fishes is only ∼1.5°C below that of fishes given a CT_Max_ test. Finally, this study raises broader questions about how *E*_O_2__ and its modulation vary between fish species and ecotypes, and is affected by environmental history, and it is hoped that this paper will inspire such research.

In addition, this study has several important implications for the field of fish thermal tolerance and the ways in which experiments examining this important aspect of fish biology are conducted. First, the present results highlight limitations of using traditional CT_Max_ tests alone as a predictor of a species' resilience or susceptibility in the face of rising temperatures (climate change). The CT_Max_ of *L. apodus* (39.7°C) is comparable to that of other tropical reef species (Illing et al., 2020; Madeira et al., 2017; Vaughan et al., 2025), yet the reduction in CTS_Max_ (∼1.7°C) suggests that thermal safety margins will be narrower when intense activity is required. Given that reef-associated fishes routinely engage in sustained swimming (activity) related to foraging, predator avoidance and territory defence, their realised upper thermal tolerance is likely to be closer to CTS_Max_ than to CT_Max_ (i.e. it is currently being overestimated by the latter protocol). Thus, thermal limits derived from resting fish may systematically underestimate the effects of increasing temperatures on fish ecology and biodiversity (Payne and Smith, 2017; Rummer and Munday, 2017). CTS_Max_, by contrast, imposes combined thermal and exercise stressors on the fish, and allows for the simultaneous assessment of performance and oxygen transport limitations (i.e. those related to *Q̇*, *f*_H_, *S*_V_ and *E*_O_2__) under active conditions that might represent the most challenging for fishes in an ecological context. Although still laboratory based, this test provides insights into how physiological systems perform under extremely demanding life functions. This approach aligns with recent calls (Sandrelli et al., 2024; Gamperl and Stevens, in press) to incorporate dynamic and multi-stressor assays into assessments of thermal tolerance.

Second, the coordinated cardiorespiratory adjustments utilised by the schoolmaster snapper to support the metabolic costs associated with large acute increases in temperature, near maximal exercise and these two challenges combined, is consistent with the general principles of the OCLTT hypothesis (Pörtner, 2010; Pörtner et al., 2017), which relates temperature-dependent performance to tissue oxygen supply. However, our data show that, in actively swimming tropical fish, *E*_O_2__ remains plastic and continues to increase even after *Q̇* plateaus. Such evidence implies that oxygen convection and diffusion both limit fish thermal tolerance in contrast to established models of the oxygen transport cascade in fishes (Farrell, 2009). Moreover, the results caution against using *f*_H_ alone as a proxy of thermal tolerance. Although *f*_H_ correlates with MMR, it does not capture the efficiency of oxygen delivery or utilisation. Furthermore, the ‘rapid screening protocol’ (*f*_H,Max_ test; Casselman et al., 2012) is being promoted by some researchers (e.g. see Gilbert et al., 2024) as a method that can be used to study cardiac thermal performance and heat tolerance in fishes. However, Sandrelli and Gamperl (2023) showed that *f*_H,Max_ as assessed using the ‘rapid screening protocol’ was well below that measured in free-swimming Atlantic salmon given a traditional CT_Max_ test (by ∼27 beats min^−1^; 17.6%) and our data indicate that this difference would be even greater if the fish were actively swimming. This casts further doubt on the validity of *f*_H,Max_ as determined using the ‘rapid screening protocol’ for predicting the thermal tolerance of fishes.

Finally, from an ecological standpoint, the findings of this study have important implications for predicting the vulnerability, or resilience of tropical reef fishes to ocean warming. Tropical marine fishes appear to already be living close to their thermal maximum, and thus, have small thermal safety margins (Rummer et al., 2014; Madeira et al., 2017; Munday et al., 2008). The lower value for CTS_Max_ versus CT_Max_ in *L. apodus* suggests that even modest increases in water temperature could push active fish beyond their functional limits, particularly during energetically demanding behaviours. In this context, species with plastic *E*_O_2__ responses or a greater capacity to upregulate *f*_H_ under warming conditions, may have an advantage.
